# Cyclacene-derived carbon lattices with distorted hexagonal tiling and in-plane π-orbitals: coexistence of flat and Dirac bands†

**DOI:** 10.1039/d5ma00055f

**Published:** 2025-07-24

**Authors:** Divanshu Gupta, Michael Mastalerz, J. Michael Gottfried, Holger F. Bettinger

**Affiliations:** a Institut für Organische Chemie, Eberhard Karls Universität Tübingen Auf der Morgenstelle 18 72076 Tübingen Germany holger.bettinger@uni-tuebingen.de; b Organisch-Chemisches Institut, Ruprecht-Karls-Universität Heidelberg, Im Neuenheimer Feld 272 69120 Heidelberg Germany; c Fachbereich Chemie, Philipps-Universität Marburg Hans-Meerwein-Str. 4 35032 Marburg Germany

## Abstract

The discovery of nanomaterials with unique electronic band structures, such as flat bands, has drawn significant interest for enabling novel physical phenomena and advanced technological applications. Kagome lattices, characterized by corner-sharing triangles, are a notable class of materials featuring the coexistence of flat and Dirac bands. This study investigates a new class of carbon lattices derived from cyclacene molecules (cyc-CL), featuring a distorted hexagonal tiling. In these two-layer carbon structures with hydrogen-saturated bonds, π orbitals lie parallel to the layers, unlike typical 2D carbon materials. Using first-principles DFT calculations, we analyze three variants (6-cyc-CL, 12-cyc-CL, and 18-cyc-CL), examining band gaps, density of states (DOS), and orbital contributions. Cyc-CL systems exhibit tunable band gaps, flat and Dirac bands near the Fermi level, and dominant π orbitals from p_*x*_ and p_*y*_ states. These results highlight cyc-CL's potential for studying quantum phenomena and enabling nanotechnology applications.

## Abstract 2

A new carbon lattice derived from 2D polymerization of *n*-cyclacenes (*n* = 6, 12, 18) is shown to have special electronic properties.

## Graphical abstract

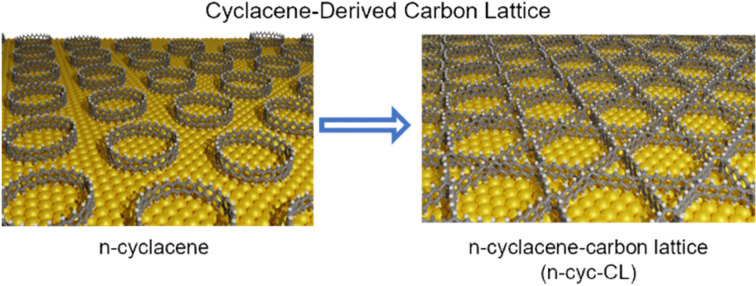

## Introduction

Over the past two decades, the design, discovery, and characterization of nanomaterials and nanostructures with unique electronic band structure features have gained tremendous attention. Among these, materials and structures exhibiting dispersionless electronic states, or flat bands, have been widely studied because electrons in these states exhibit suppressed kinetic energy, resulting in spatial localization, which in turn leads to intriguing physical phenomena and applications in energy storage, optoelectronics, and quantum computing.^1–5^

**Fig. 1:**
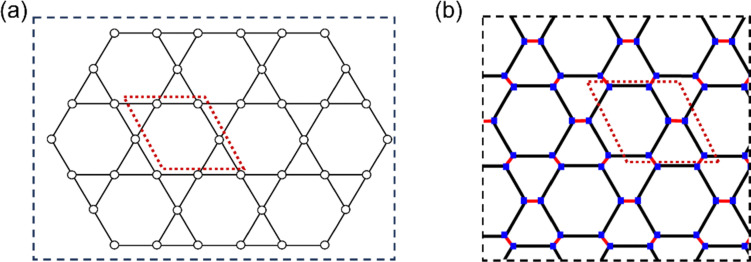
(a) Schematic representation of a Kagome lattice (trihexagonal tiling, symmetry group *632). (b) Schematic representation of *n*-cyc-CL derived distorted hexagonal tiling (symmetry group *632). The dotted lines represent the unit cells of the lattice and structures.

Kagome compounds, particularly in two-dimensional (2D) layered forms, have emerged as a key area of research in materials science due to their unique lattice structure and electronic properties.^6–14^ The Kagome lattice (Fig. 1a), originally named after a Japanese basket-weaving pattern, is a 2D network composed of corner-sharing triangles arranged in a hexagonal framework.^15^ In the physics literature, these lattices are often identified as a “decorated honeycomb” or “star lattice” structure.^16–23^ This geometric configuration imparts Kagome materials with “frustration”—a phenomenon where electrons or spins in the lattice cannot simultaneously satisfy all favorable interactions. This characteristic, combined with the lattice's symmetry, gives rise to unconventional and desirable electronic states, including flat bands and Dirac bands.^24–26^

The remarkable electronic behavior of Kagome compounds is rooted in geometric frustration, which induces flat bands in their electronic band structure. Flat bands are formed when the kinetic energy of electrons is effectively quenched, leading to highly localized electronic states. These flat bands are especially significant because they confine electrons within an extremely narrow energy range, leading to a remarkably high density of states (DOS). This characteristic has been linked to various phenomena like Wigner crystallization,^27,28^ flat-band ferromagnetism,^29^ fractional quantum Hall states,^30–34^ and a possibility of high-temperature superconductivity.^1,35,36^ These properties render such materials highly promising for various energy, quantum computing, and optoelectronics applications. Flat bands have been observed in several Kagome-based 2D materials, especially in systems based on transition metals or carbon.^7–9^ In such systems, destructive interference in the hopping processes causes the single-particle eigenstates to localize as degenerate states on different plaquettes.^10^ The Bloch-wave band states formed are superpositions of these localized states, resulting in effectively dispersionless bands.^10^

In addition to flat bands, Kagome structures often feature Dirac bands, another hallmark of their electronic architecture. Dirac bands are characterized by linear energy-momentum dispersion near certain points in the Brillouin zone, supporting electrons that behave as massless Dirac fermions. This behavior results in high mobility and low energy dissipation.^37^ The Dirac bands in Kagome lattices emerge from the lattice's symmetry and unique geometric features, which create quasi-relativistic electron behaviors akin to those observed in graphene.^9^ This coexistence of flat and dispersive Dirac bands enables simultaneous exploration of highly localized and delocalized electronic states, a unique feature that has sparked interest in their potential use in electronic devices.^8,38–40^

Recent computational studies have focused intensively on 2D and bulk carbon allotropes that exhibit unique electronic features, such as Dirac cones, flat bands, and non-trivial topological states.^7,41–47^ However, while various 1D carbon allotropes are well-documented, these structures typically lack associations with flat band physics.^48–53^ Only a few studies have explored the potential for flat bands in 1D carbon nanomaterials. Rare instances of flat bands have been observed in systems such as partially flat bands in zigzag graphene nanoribbons,^54^ spin-polarized flat bands in hydrogenated carbon nanotubes,^55^ and moiré flat bands in certain chiral carbon nanotubes with collapsed structures^56,57^ or incommensurate double-walled configurations.^58^

In light of this, our study aims to fill an existing gap by examining a family of realistic carbon nanostructures that naturally host flat bands across their Brillouin zones. Unlike prior approaches, which often rely on doping or structural instabilities to induce flat bands, the flat electronic states in our structures emerge due to inherent geometric and orbital frustration. Specifically, we focus on a cyclacene-derived carbon lattice (cyc-CL). A cyc-CL features a type of distorted hexagonal tiling (see Fig. 1b). It shares the symmetry group *632 with the regular hexagonal tiling of the graphene lattice, but it differs from it by being composed of regular and irregular hexagons. A unique feature of the cyc-CL lattice, which consists of two layers of carbon atoms and has hydrogen saturation to avoid dangling bonds, is that the π orbitals are not oriented orthogonal to the layer, but rather parallel to the layer. To the best of our knowledge, the chemical characteristics and electronic properties of such materials remain unexplored.

Cyclacenes (C_4*n*_H_2*n*_) are unique carbon-based molecules, which have captured significant interest with their hoop-shaped, zigzag carbon frameworks similar to zig-zag carbon nanotubes.^59–61^ Initially conceptualized by Heilbronner in 1954, cyclacenes belong to the polycyclic aromatic hydrocarbon family and show potential for use in organic semiconductors due to their tunable electronic properties.^62–64^ However, their synthesis is challenging due to high structural strain and reactivity.^65–69^ Noteworthy efforts include work by Stoddart *et al.*,^64,70–73^ Cory *et al.*,^74,75^ and Schlüter *et al.*^76,77^ on synthesizing specific cyclacene sizes, while recent advances by Itami *et al.* in carbon nanobelts offer promising synthetic insights.^78,79^ Wang's team reported forming [8]-cyclacene *via* a retro-Diels–Alder reaction in the mass spectrometer,^80^ while attempts to generate [12]-cyclacene on surfaces by Gross, Peña *et al.* have thus far been unsuccessful.^81^

**Fig. 2:**
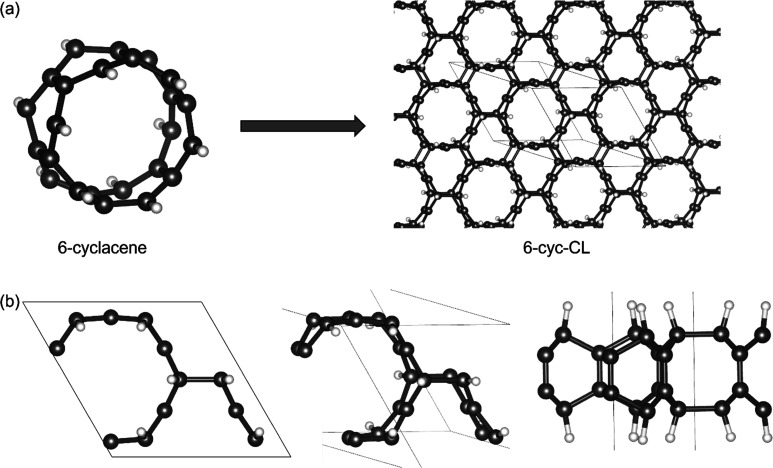
(a) Schematic representation of the assembly of cyclacene into carbon lattices of distorted hexagonal tiling (*n*-cyc-CL), along with black lines representing the unit cells of the structures. (b) Top view (left), perspective view (middle), and side view (right) of a unit cell of 6-cyc-CL.

More recently, a computational study of a crystal composed of cyclacene molecules as building block was reported.^82^ A two-dimensional crystal of [6]-cyc molecules held together by weak intermolecular forces forms through a self-assembly process, resulting in a structure referred to as [*n*]crystacene.^82^ In this arrangement, the molecules organize into a triangular lattice with their tube axes oriented perpendicular to the crystal plane. The investigation of the electronic structure of [6]crystacene explored the development of molecular local moments, the characteristics of intermolecular exchange interactions, and their dependence on the rotational angle.^82^ Recently, a computational investigation of cyclacene dimerization revealed this to be a highly exothermic and low barrier process.^83,84^ This suggests that the two-dimensional [6]crystacene array is unstable towards collapse into a covalent framework that resembles a cyclacene-derived carbon lattice (cyc-CL) (see Fig. 2a).

Building on these studies, our work examines the potential for cyclacene molecules to assemble into carbon lattices of distorted hexagonal tiling, driven by their high reactivity and dimerization energies (see Fig. 2a). This is a conceptually novel type of carbon lattice as it involves π orbitals that lie within the two-dimensional lattice and not orthogonal to it. This work marks the first investigation into the electronic structure of cyclacene-derived carbon lattices (cyc-CL), focusing on their intrinsic flat-band features and the underlying geometric and orbital frustration. It paves the way for understanding the unique electronic properties and exploring the potential applications of cyc-CL in nanotechnology and quantum materials.

## Computational methods

We performed first-principles calculations using density functional theory (DFT) as implemented in the VASP code.^85–87^ The potential of the core electrons and the exchange–correlation interaction between the valence electrons are described by the projector augmented wave^88,89^ and the generalized gradient approximation (GGA) of Perdew, Burke, and Ernzerhof (PBE).^90,91^ A kinetic energy cutoff of 850 eV was employed. The atomic positions were optimized using the conjugate gradient method, with an energy convergence criterion of 10^−7^ eV between two consecutive steps. A maximum force of 10^−3^ eV Å^−1^ was allowed on each atom. To mitigate spurious interactions, symmetric monolayers were placed in the middle of crystals and separated by vacuum layers of 15 Å at both the top and bottom. Integrations over the Brillouin zone were performed using a 7 × 7 × 1 Gamma-centered *k*-point grid for structural relaxations. In contrast, a finer grid 18 × 18 × 1 was used to calculate the electronic density of states (DOS) of 6-cyc-CL, 12-cyc-CL, and 18-cyc-CL, respectively and the results were post-processed using the VASPKIT package.^92^ Since the PBE functional underestimates the band gap, we used the Heyd–Scuseria–Ernzerhof (HSE06)^93^ functional (with a screening parameter of 0.2 Å^−1^ and a mixing parameter of 0.25) to obtain accurate electronic band structures, for which a kinetic energy cutoff of 520 eV was employed. For HSE06, *k*-point mesh was generated using VASPKIT.^92^ The resolution values of the normal weighted *k*-mesh and zero-weighted *k*-path were set to 0.025 and 0.040 for 6-cyc-CL, 0.025 and 0.030 for 12-cyc-CL, and 0.020 and 0.040 for 18-cyc-CL, respectively. The *k*-mesh for the self-consistent field (SCF) calculations was 9 × 9 × 1, 4 × 4 × 1, and 3 × 3 × 1 for 6-cyc-CL, 12-cyc-CL, and 18-cyc-CL, respectively. The number of *k*-points along each line of the *k*-path was as follows: Γ → M: 12, 10, 7; M → K: 7, 5, 5; K → Γ: 14, 11, 8 for 6-cyc-CL, 12-cyc-CL, and 18-cyc-CL, respectively. To study the dynamic stability of *n*-cyc-CL, phonon dispersion calculations were performed using the Phonopy code^94,95^ in conjugation with DFPT (density-functional perturbation theory),^96,97^ as implemented in VASP. Subsequently, *ab initio* molecular dynamics (AIMD) simulations were conducted at temperatures of 300 K and 500 K, employing the Nosé–Hoover thermostat.^98,99^ A time step of 1.0 fs was used for integration during simulations.

## Results and discussion

**Fig. 3:**
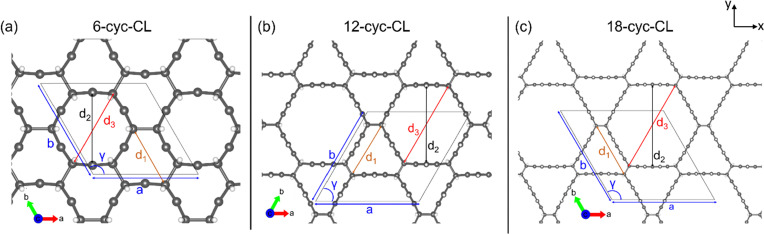
(a)–(c) Top view of atomic structures for *n*-cyc-CL (*n* = 6, 12, 18 respectively), along with black lines representing the unit cells of the structures.

The structures of the cyc-CL that we propose (Fig. 3a-c) are termed *n*-cyc-CL (*n* = 6, 12, 18) where *n* denotes the number of benzene rings in the cyclacene units that form the lattice by two-dimensional polymerization. The space group of the cyc-CL family is *P*1. The optimized lattice parameters for 6-cyc-CL, 12-cyc-CL, and 18-cyc-CL are *a* = *b* = 7.004 Å, 11.866 Å, and 16.772 Å, respectively. The lattice angles for 6-cyc-CL, 12-cyc-CL, and 18-cyc-CL are *γ* = 120°, 60°, 120°, respectively. The coordinates for each structure are presented in the ESI.†

To get further insight into the geometrical parameters, the pore diameters for equilateral hexagonal and distorted hexagonal holes in the *n*-cyc-CL structures (as shown in Fig. 3a–c) were investigated using the GGA-PBE method. The diameter of the distorted hexagonal hole (*d*_1_) varies among the 6-cyc-CL, 12-cyc-CL, and 18-cyc-CL structures, measuring 4.314 Å, 6.745 Å, and 9.198 Å, respectively. For the equilateral hexagonal holes, the diameters *d*_2_ (4.858 Å, 9.084 Å and 13.410 Å) and *d*_3_ (5.378 Å, 10.250 Å and 15.159 Å) also vary across the three structures.

The formation energy was calculated using the GGA-PBE method and is defined as: 
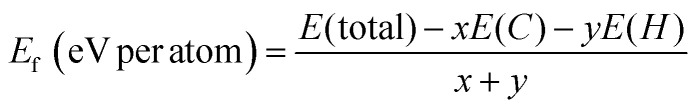
, where *E*_(total)_ is the total energy of the system, *E*_(C)_ and *E*_(H)_ are the ground state energies of single carbon and hydrogen atoms, respectively, and *x* and *y* represent the number of carbon and hydrogen atoms, respectively.

The calculated formation energies *E*_f_ (eV per atom) for 6-cyc-CL, 12-cyc-CL, and 18-cyc-CL are 0.211, 0.062, and 0.038, respectively. The positive values of *E*_f_ are consistent with previous studies, which report positive formation energies at low hydrogen concentrations.^100–103^ For comparison, the formation energy per carbon atom in various (10,0) carbon nanotube caps ranges from 0.370 eV per atom (for structures containing 40 carbon atoms) to 0.290 eV per atom (for 60 carbon atoms).^104^ Additionally, reported values for the (10,0) carbon nanotube and C_70_ are 0.137 eV per atom and 0.376 eV per atom, respectively.^104^ A tight-binding calculation on C_60_ yields a formation energy of approximately 0.40 eV per atom.^105,106^ Furthermore, the phonon band spectrum (see Fig. S11, ESI†) shows no imaginary frequency, confirming the dynamic stability of *n*-cyc-CL structures. To assess the finite-temperature structural stability of *n*-cyc-CL, *ab initio* molecular dynamics (AIMD) simulations were performed at 300 K and 500 K. Throughout the duration of these simulations, the total energy of the system remained stable (Fig. S12, ESI†), indicating no significant structural distortions. These results suggest that *n*-cyc-CL retains its structural integrity at and above room temperature, supporting its viability as a physically realistic two-dimensional material.

### Electronic properties

**Fig. 4:**
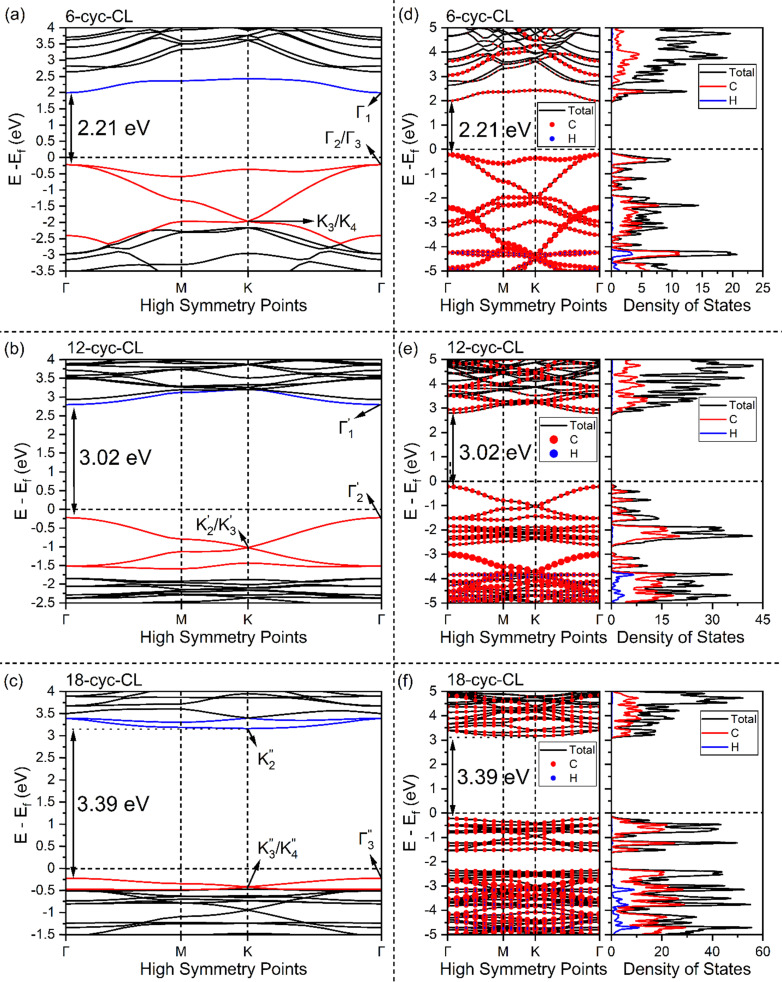
The electronic band structures for (a) 6-cyc-CL, (b) 12-cyc-CL, and (c) 18-cyc-CL, respectively. The labels Γ_1_ and 
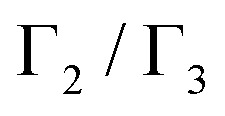
 in (a) represent CBM (conduction band minimum) and VBM (valence band maximum) orbital frustration states in 6-cyc-CL, while 
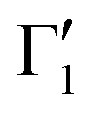
, and 
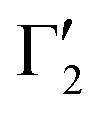
 in (b) and 
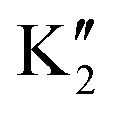
, and 
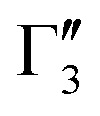
 in (c) represent CBM and VBM in 12-cyc-CL, and 18-cyc-CL, respectively. 
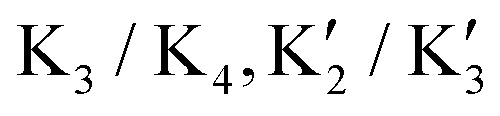
, and 
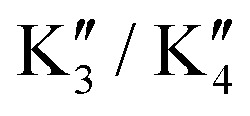
 in (a), (b), and (c) labels Dirac points in 6-cyc-CL, 12-cyc-CL, and 18-cyc-CL, respectively. Atom-projected band structure and DOS of (d) 6-cyc-CL, (e) 12-cyc-CL, and (f) 18-cyc-CL, respectively.

The band structures for the *n*-cyc-CL (*n* = 6, 12, 18) focus on their in-plane electronic properties along the *k*-path Γ–M–K–Γ (cyc-CL lattice plane in real space). The band structures change significantly with the variations in the structures. To clearly illustrate the evolution of the bands with these structural changes, red and blue curves highlight the valence bands and conduction bands around the Fermi level, respectively (Fig. 4a–c). The band structures show that 6-cyc-CL and 12-cyc-CL exhibit a direct band gap, while 18-cyc-CL has an indirect band gap. The bands display large dispersions, indicating that the electrons possess small effective masses in every direction.

In the band structure of 6-cyc-CL, there is a flat band around the Fermi level, which is common in systems with geometries such as Kagome lattice, Lieb lattice, or other related structures.^107^ This suggests the presence of local single-particle states as the eigenstates of the band Hamiltonian. The flat band in 6-cyc-CL disappears around the Fermi level and shifts downward in 12-cyc-CL and 18-cyc-CL band structures. The lowest conduction band of *n*-cyc-CL structures, indicated by blue bands, is observed to shift upwards from 6-cyc-CL to 18-cyc-CL. However, Dirac points at the high symmetry point K are observed for all the cyc-CL structures around the Fermi level.

For 6-cyc-CL, a flat band and a Dirac band coexist at the Fermi level. This resembles the coexistence of flat band and steep band discussed in earlier studies,^7,108^ which investigated their potential applications in superconductivity. The band gap of *n*-cyc-CL (ranging from 2.2 eV to 3.4 eV) is considerably smaller than that of diamond (5.3 eV)^109^ and graphane (4.4 eV)^110^ at the same computational level (HSE06), but it remains relatively large.

To better understand the atomic contribution of *n*-cyclacenes in the electronic structure of *n*-cyc-CL, we calculated the atom-projected band structure, and density of states (DOS) using both GGA (see Fig. S1–S9 in ESI†) and hybrid functionals (Fig. 4d–f). The valence bands show a greater contribution from carbon atoms around the Fermi level, while the hydrogen atom character is more prominent in the lower energy valence bands. In the conduction bands, no hydrogen atom character is observed. Furthermore, atom-specific projected band structures and DOS using hybrid functional (see Fig. S10, ESI†) show that the carbon atoms, which result from the rung bonds (for 6-cyc-CL) or the benzene (for 12-cyc-CL) and naphthalene (for 18-cyc-CL) units of the cyclacene building blocks, play a significant role in forming the flat and Dirac bands near the Fermi level.

**Fig. 5:**
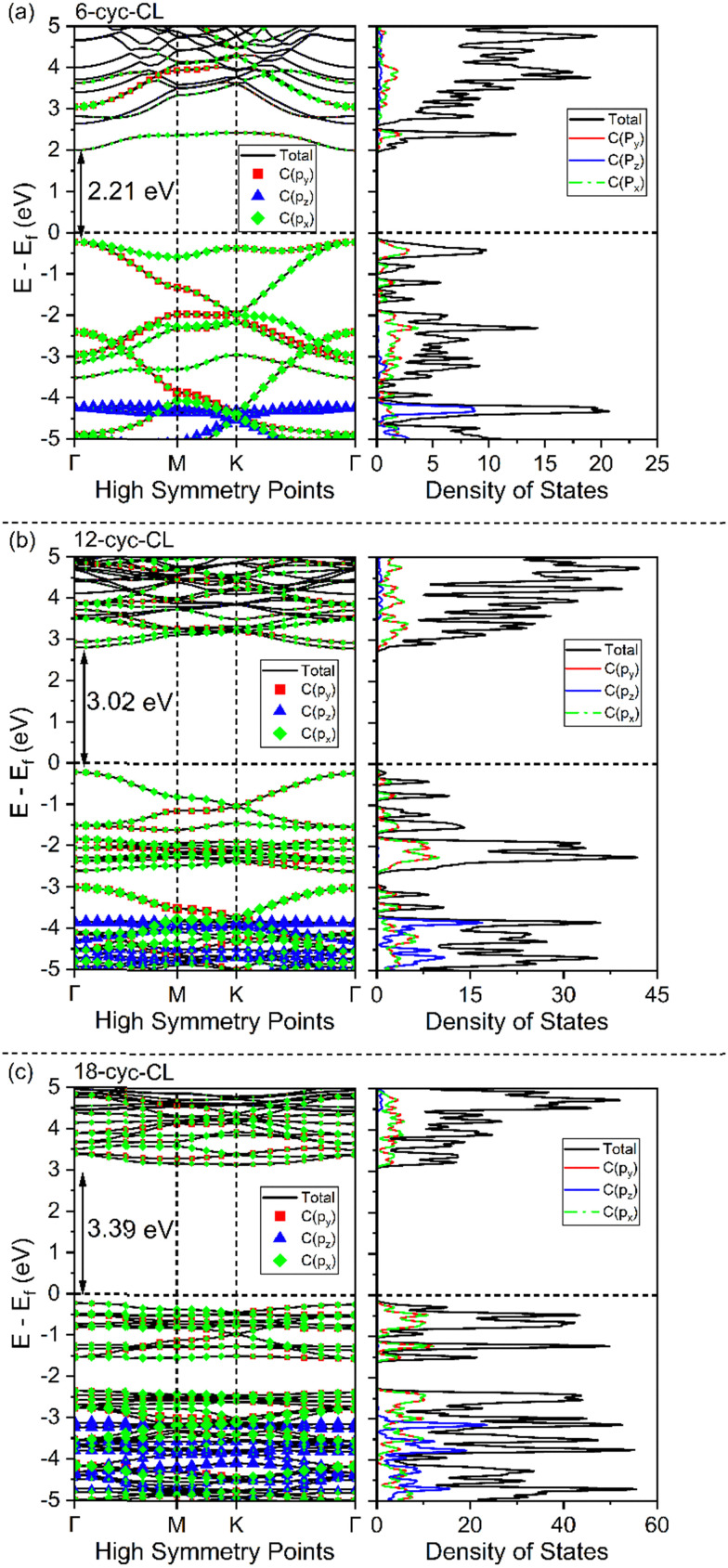
Orbital projected band structure and DOS of (a) 6-cyc-CL, (b) 12-cyc-CL, and (c) 18-cyc-CL.

To gain a better understanding of the nature of the bonds in the *n*-cyc-CL, the density of states (DOS) is projected onto the orbitals of carbon atoms for HSE06 computations (Fig. 5). Note that the *xy* plane is perpendicular to the cyclacene ring, thus the p_*x*_ and p_*y*_ orbitals here are the π orbitals of the *n*-cyclacenes. It is found that the projected DOS (PDOS) around the Fermi level (−3 to 3 eV) is mainly attributed to the electrons of p_*x*_ and p_*y*_ orbitals. There is no contribution from the s and p_*z*_ orbitals to the total electronic states at the Fermi level. However, at the lower energies, the contribution of p_*z*_ orbitals becomes more significant.

The dispersionless states observed in *n*-cyc-CL arise from destructive interference caused by geometric and orbital frustration, a phenomenon also reported in Kagome lattice systems.^31,111,112^ At the flat band, electrons localize, giving rise to a pronounced peak in the electronic DOS near the Fermi level, as illustrated in Fig. 4 and 5. It is also important to highlight certain distinctions between the electronic properties of cyc-CL and those of carbon Kagome nanotubes (CKNTs) or their conventional analogs.^10,24^ Unlike traditional CKNTs, the intriguing electronic behavior of cyc-CL is primarily linked to π electrons originating from the p_*x*_ and p_*y*_ orbitals, whereas the p_*z*_ orbitals remain mostly inactive, as confirmed by the PDOS data presented in Fig. 5.

**Fig. 6:**
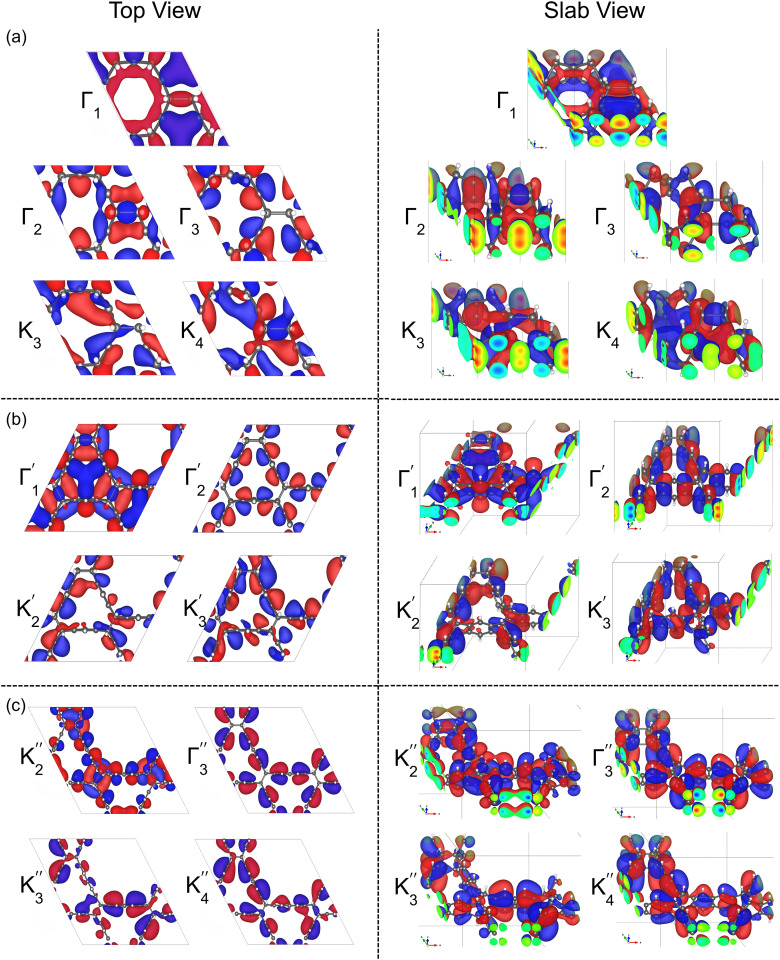
Wavefunctions are shown in both top view (left) and slab view (right) for the states (a) Γ_1_, Γ_2_/Γ_3_ and K_3_/K_4_ of 6-cyc-CL (b) 
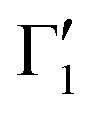
, 
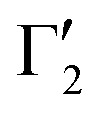
 and 
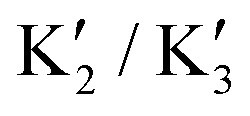
 of 12-cyc-CL, and (c) 
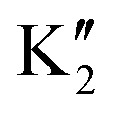
, 
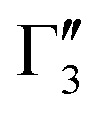
 and 
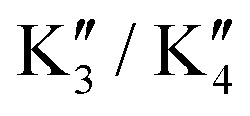
 of 18-cyc-CL corresponding to the states labelled by arrows in Fig. 4(a)–(c).

As seen from the PDOS, the band edges of the *n*-cyc-CL structures are primarily dominated by π orbitals, which conveniently explains the relationship between the structures and their electronic properties. In 6-cyc-CL, a singlet state at *Γ*_1_, which is the conduction band minimum (CBM), exhibits anti-bonding character between the layers in the distorted hexagonal ring, as shown in Fig. 6. Similarly, in the case of 12-cyc-CL, the CBM at 
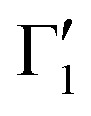
 is a singlet state with an antibonding character between the layers in the distorted hexagonal ring. In the case of 18-cyc-CL, the CBM at 
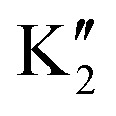
 is a mixed state with both bonding and anti-bonding character.

The valence band maximum (VBM) is a doublet state, represented by Γ_2_ and Γ_3_ for 6-cyc-CL. In contrast, for 12-cyc-CL and 18-cyc-CL, the VBM is a singlet state, represented by 
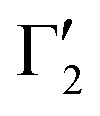
 and 
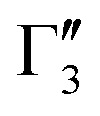
, respectively, both exhibiting π-bonding character. For the Dirac points, K_3_ and K_4_, the electrons are delocalized across the entire unit cell, particularly at the lattice joints, indicating strong interactions between lattice sites. In contrast, the Dirac points 
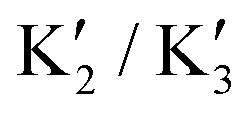
, and 
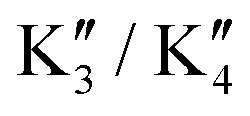
 in 12-cyc-CL and 18-cyc-CL, respectively, show weak interactions between lattice sites, as the electrons are tightly confined within the π orbitals of the cyclacenes. This indicates that Dirac electron channels in 12-cyc-CL and 18-cyc-CL are more aligned along the π orbitals of the cyclacenes.

## Conclusion

In this study, we introduced a novel carbon lattice structure derived from cyclacenes, termed cyclacene-derived carbon lattice (cyc-CL), and investigated its electronic properties using first-principles calculations. These structures have a distorted hexagonal tiling. *Ab initio* molecular dynamics (AIMD) simulations of *n*-cyc-CL (*n* = 6, 12, 18) demonstrate that these structures retain their overall structural integrity at and above room temperature (300 K and 500 K). Additionally, the absence of imaginary frequencies in the phonon band spectra confirms the dynamic stability of the *n*-cyc-CL systems. They exhibit dispersionless electronic states, or flat bands, alongside Dirac points around the Fermi level in their band structure and density of states.

From 6-cyc-CL to 18-cyc-CL, the flat band near the Fermi level shifts downward, while the lowest conduction band shifts upward, leading to an increased band gap across the series. Dirac points at the high symmetry point K are observed for all the cyc-CL structures around the Fermi level. Orbital-projected band structure and orbital-projected density of states analyses reveal that the p_*x*_ and p_*y*_ orbitals of carbon atoms have higher contributions around the Fermi level, surpassing the influence of the s and p_*z*_ orbitals.

The unique coexistence of flat and Dirac bands at the Fermi level in cyc-CL structures underscores their potential for exploring strongly correlated electronic phenomena in nanomaterials, positioning them as promising candidates for applications in superconductivity.

## Conflicts of interest

There are no conflicts to declare.

## Supplementary Material

MA-006-D5MA00055F-s001

## Data Availability

The data supporting this article have been included as part of the ESI.†
