# Supplementary material for: Cyclacene-derived carbon lattices with distorted hexagonal tiling and in-plane π-orbitals: coexistence of flat and Dirac bands
Source: Mater Adv. 2025 Jul 24;6(16):5439–48. doi: 10.1039/d5ma00055f (PMC12288019; doi:10.1039/d5ma00055f)
Supplement: MA-006-D5MA00055F-s001 [file MA-006-D5MA00055F-s001.pdf]

Supplementary Information (SI) for Materials Advances.
This journal is © The Royal Society of Chemistry 2025

Supporting Information

Cyclacene-Derived Carbon Lattices with Distorted Hexagonal Tiling and In-plane π-Orbitals: Coexistence of Flat and Dirac Bands

Divanshu Gupta [a], Michael Mastalerz [b], J. Michael Gottfried [c], Holger F. Bettinger [a*]

[a] *Institut für Organische Chemie, Eberhard-Karls-Universität Tübingen, Auf der Morgenstelle 18, 72076 Tübingen, Germany*

[b] *Organisch-Chemisches Institut, Ruprecht-Karls-Universität Heidelberg, Im Neuenheimer Feld 272, 69120 Heidelberg, Germany*

[c] *Fachbereich Chemie, Philipps-Universität Marburg, Hans-Meerwein-Str. 4, 35032 Marburg, Germany*

* E-Mail: holger.bettinger@uni-tuebingen.de

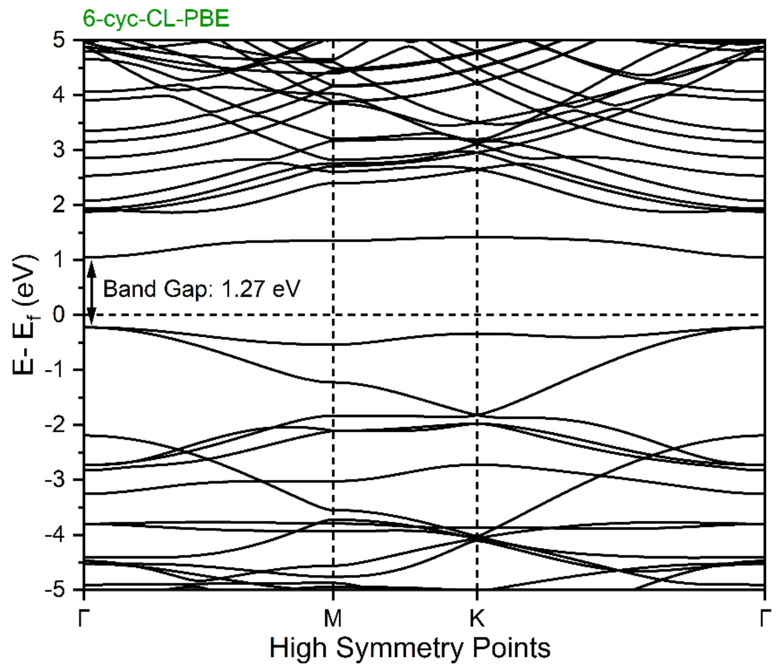

Figure S1. Electronic band structure of 6-cyc-CL calculated using GGA functional.

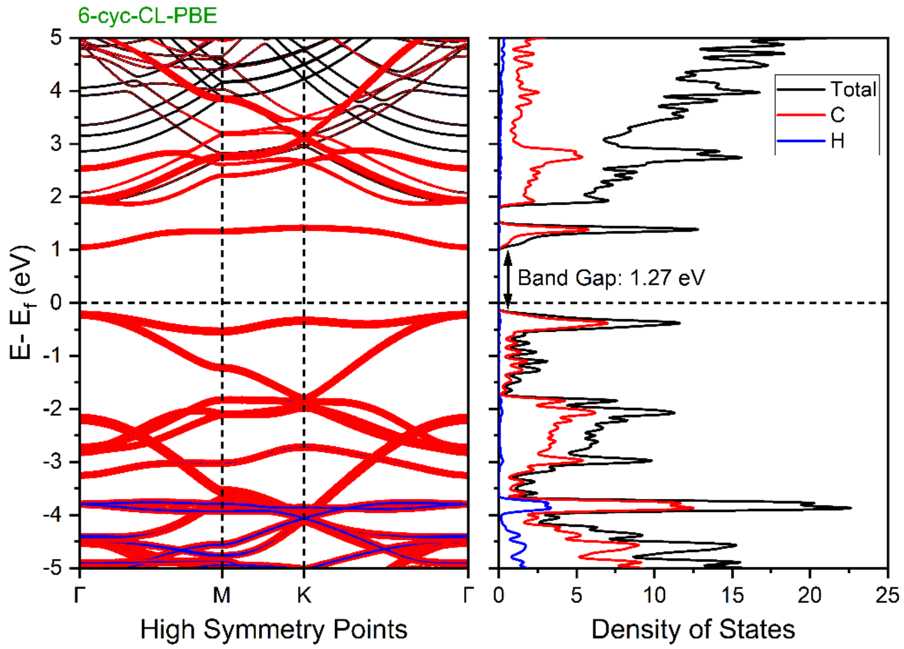

Figure S2. Atoms projected band structure and density of states (DOS) of 6-cyc-CL calculated using GGA functional.

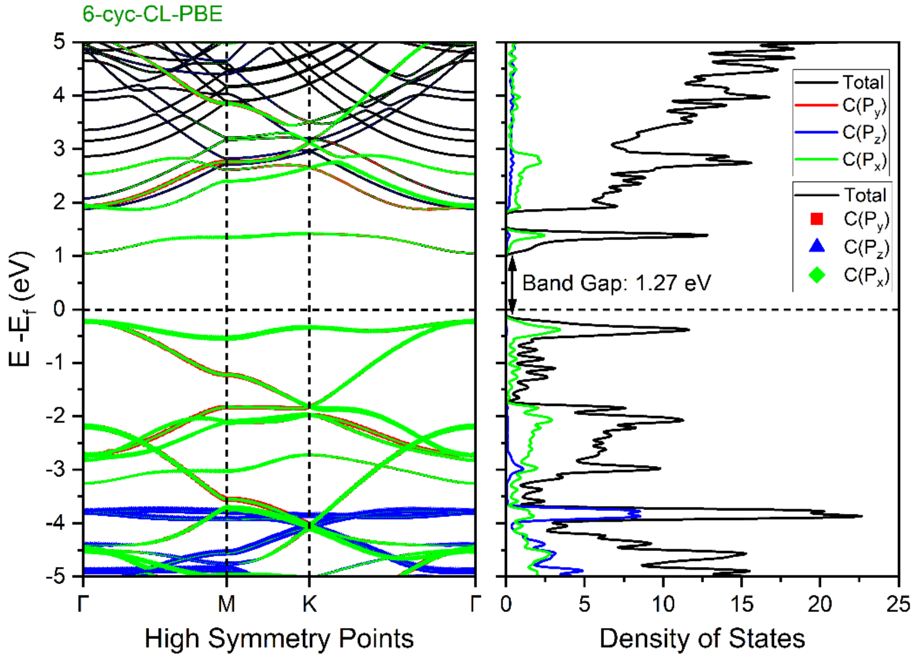

Figure S3. Orbitals projected band structure and density of states (DOS) of 6-cyc-CL calculated using GGA functional.

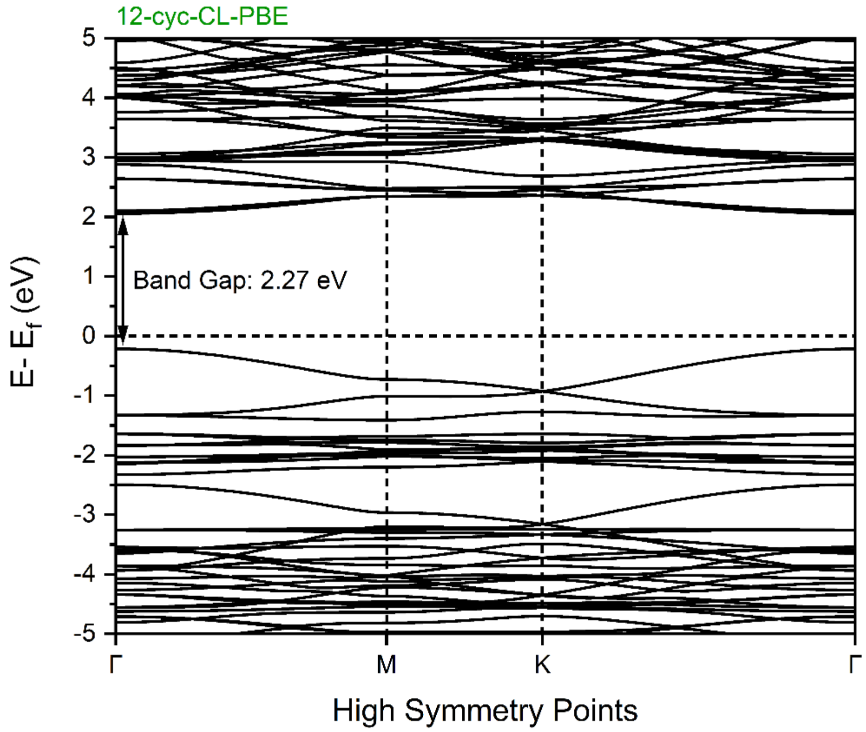

Figure S4. Electronic band structure of 12-cyc-CL calculated using GGA functional.

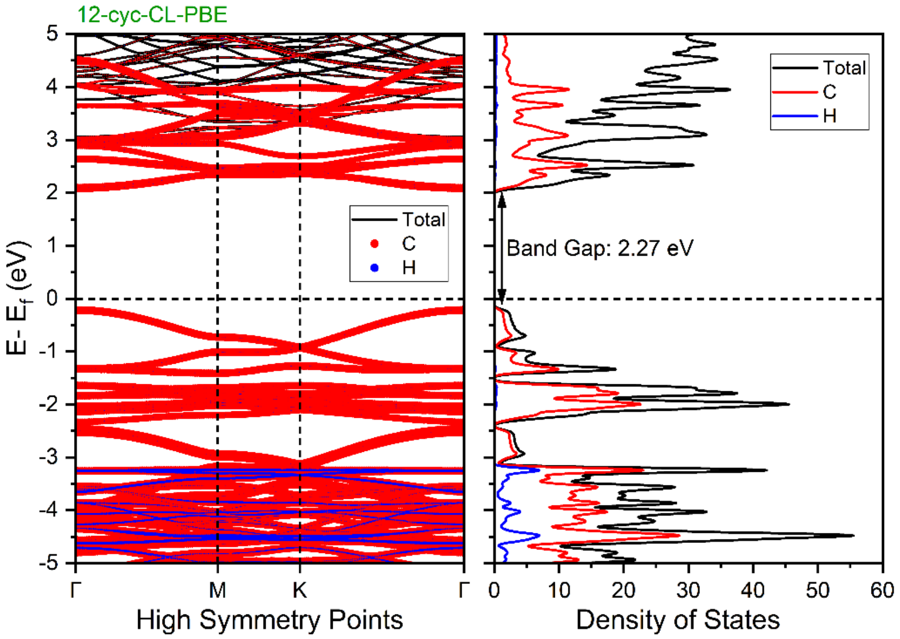

Figure S5. Atoms projected band structure and density of states (DOS) of 12-cyc-CL calculated using GGA functional.

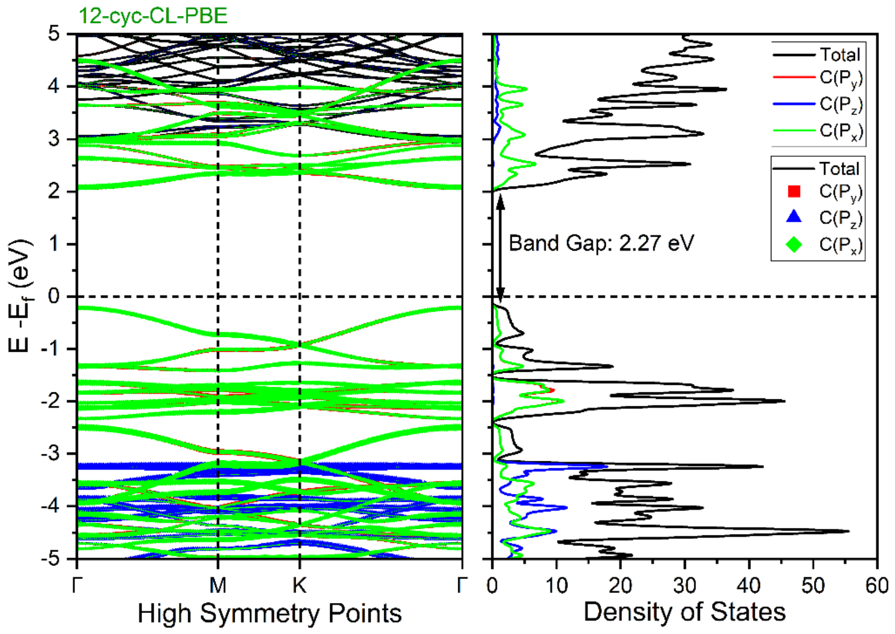

Figure S6. Orbitals projected band structure and density of states (DOS) of 12-cyc-CL calculated using GGA functional.

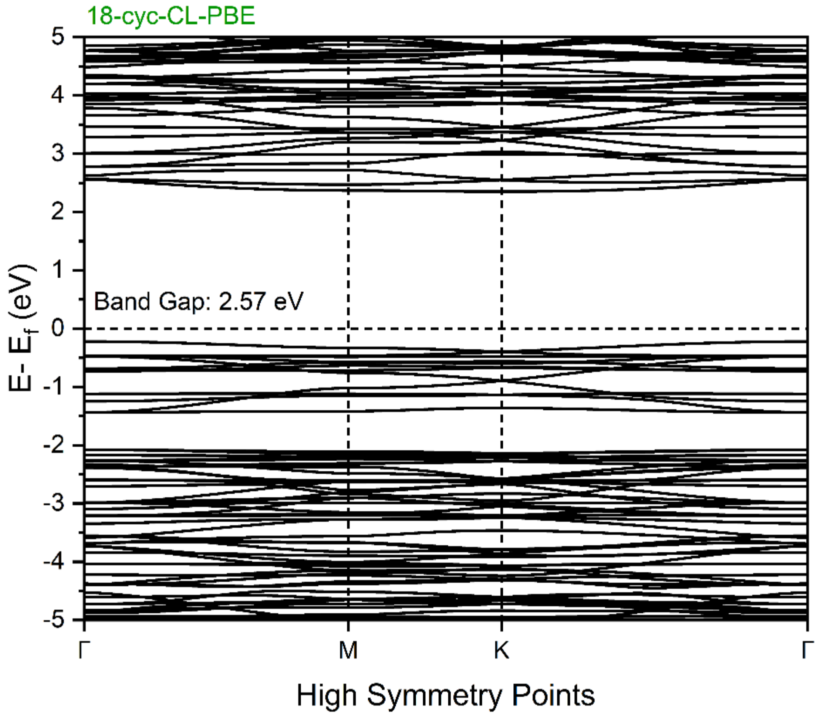

Figure S7. Electronic band structure of 18-cyc-CL calculated using GGA functional.

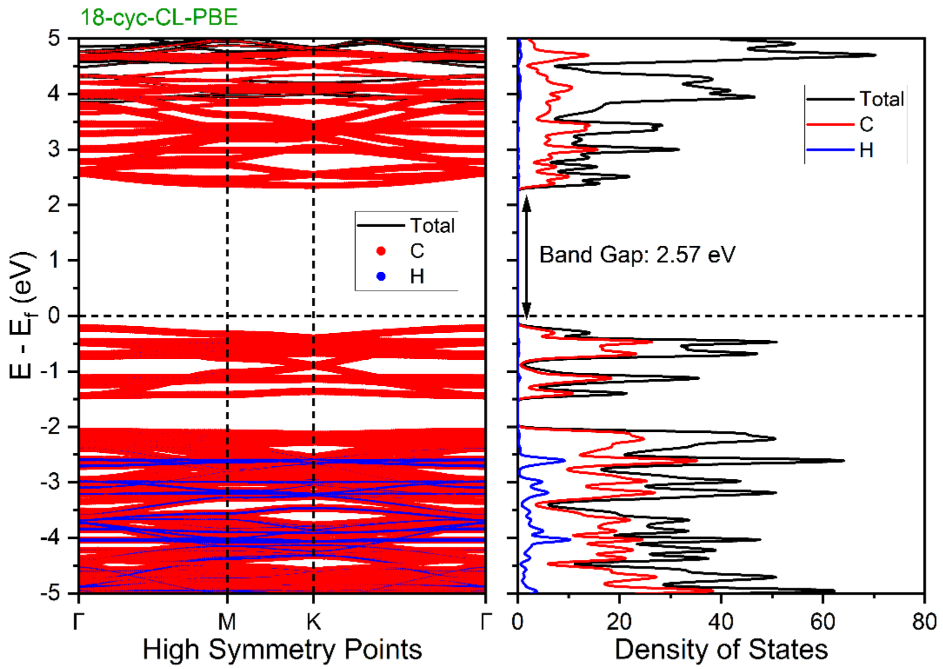

Figure S8. Atoms projected band structure and density of states (DOS) of 18-cyc-CL calculated using GGA functional.

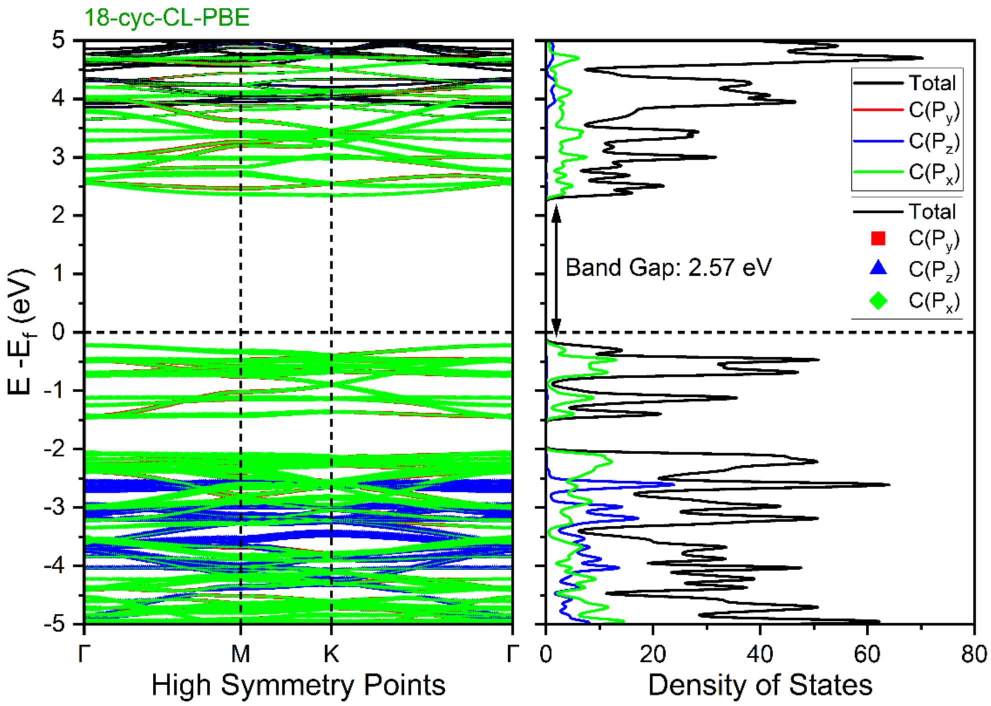

Figure S9. Orbitals projected band structure and density of states (DOS) of 18-cyc-CL calculated using GGA functional.

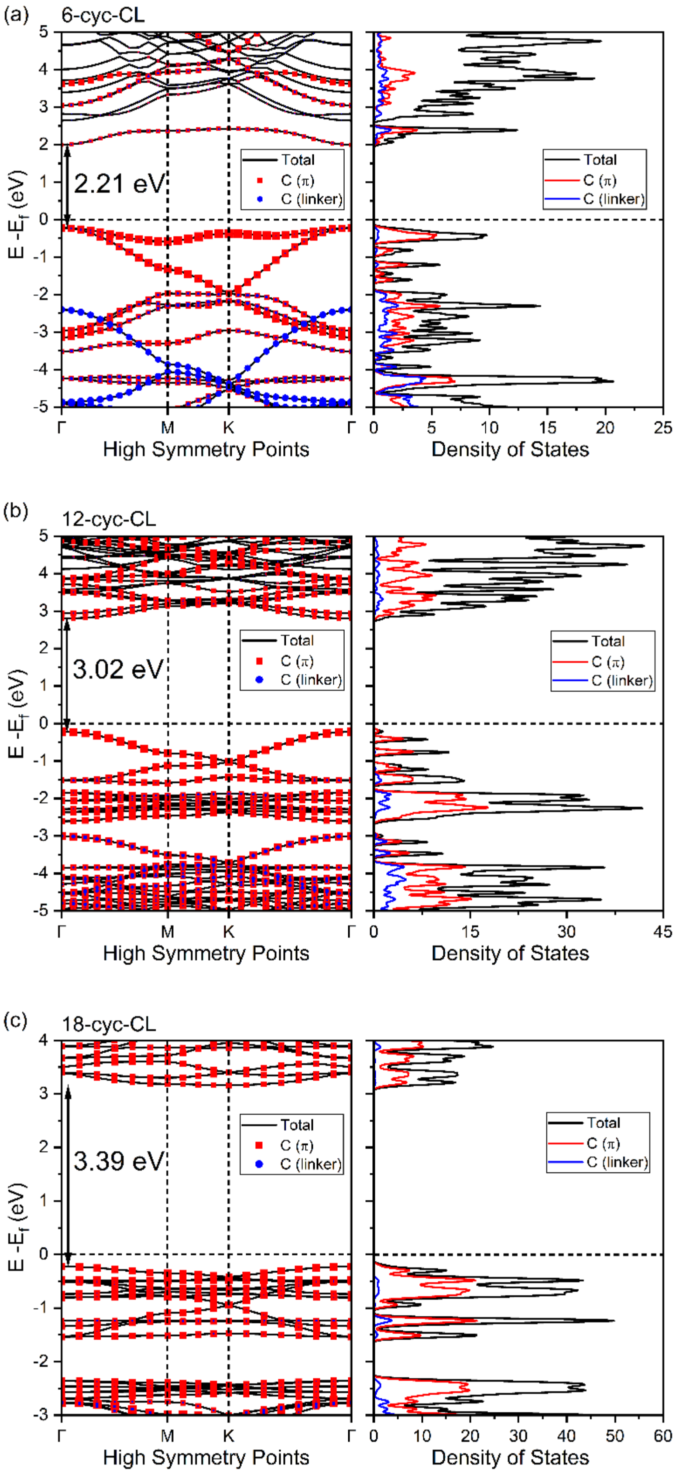

Figure S10. Carbon atom-specific projected band structure and DOS of (a) 6-cyc-CL, (b) 12-cyc-CL, and (c) 18-cyc-CL calculated using hybrid functional. C(π) are the carbon atoms which result from the rung bonds (for 6-cyc-CL) or the benzene (for 12-cyc-CL)

and naphthalene (for 18-cyc-CL) units of the cyclacene building blocks. C (linker) are the carbon atoms which are involved in the formation of the cyc-CL.

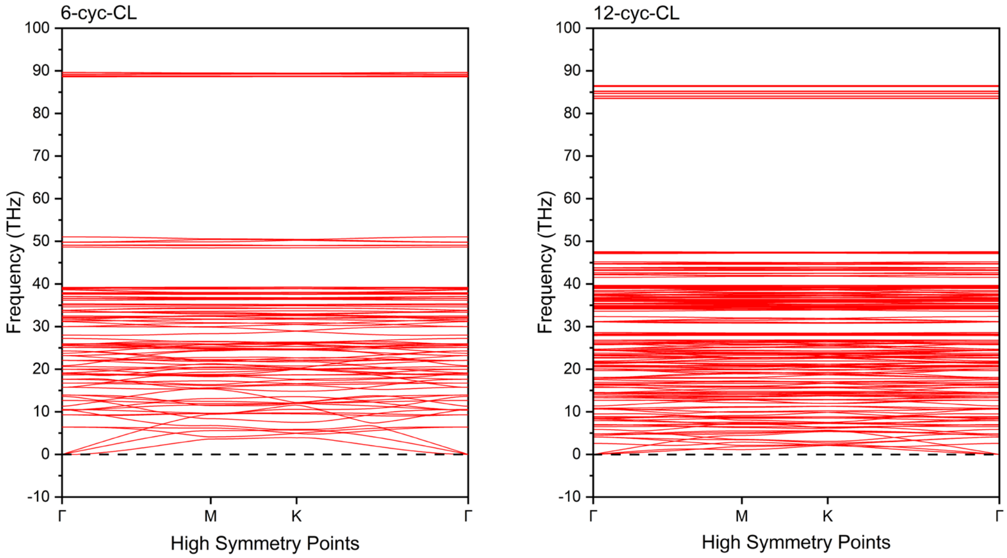

Figure S11. Phonon spectra of (left) 6-cyc-CL and (right) 12-cyc-CL calculated using GGA functional.

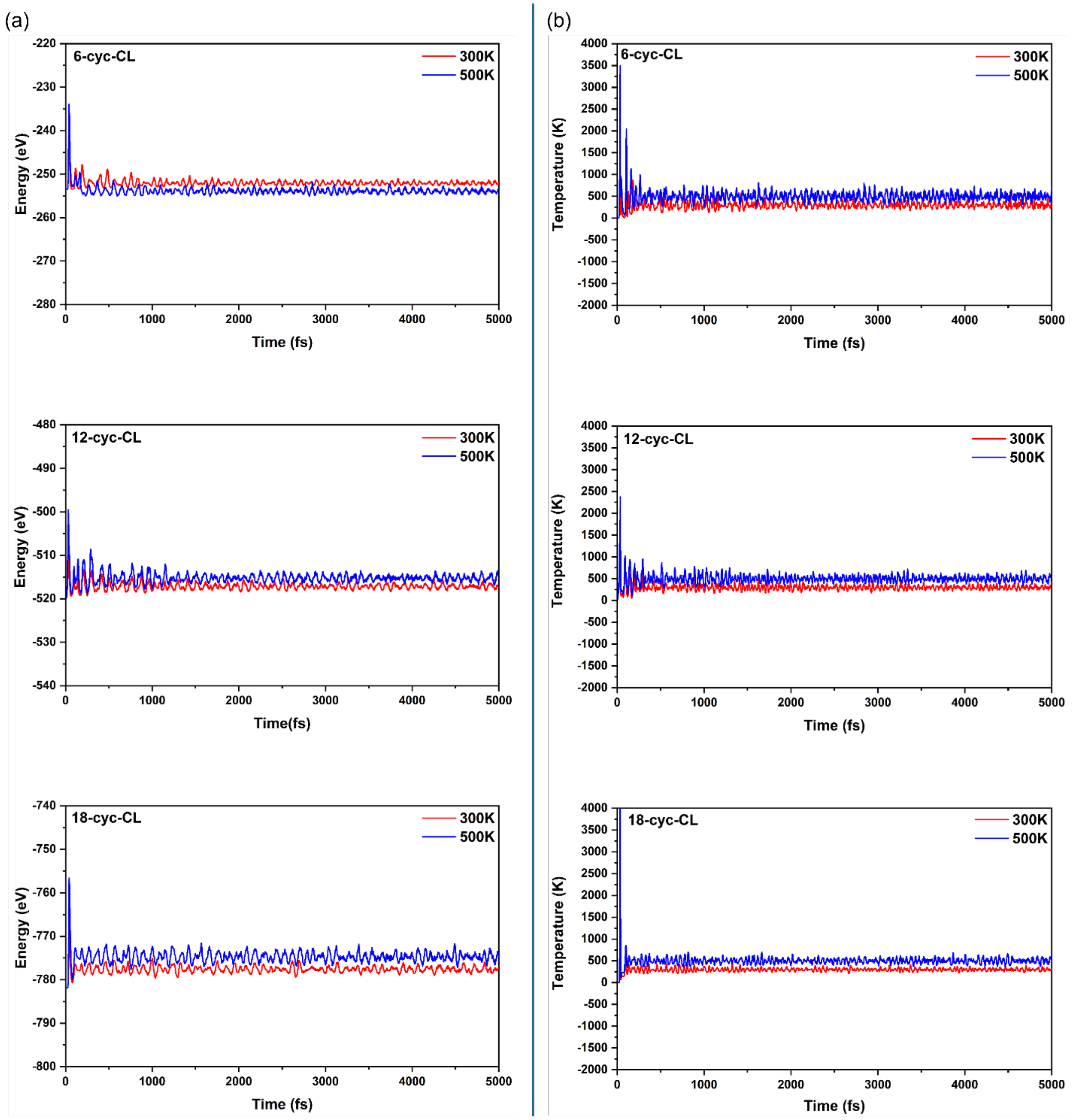

Figure S12. AIMD simulation for the (a) energy fluctuation and (b) temperature fluctuation at T = 300 and 500 K.

(a)

(b)

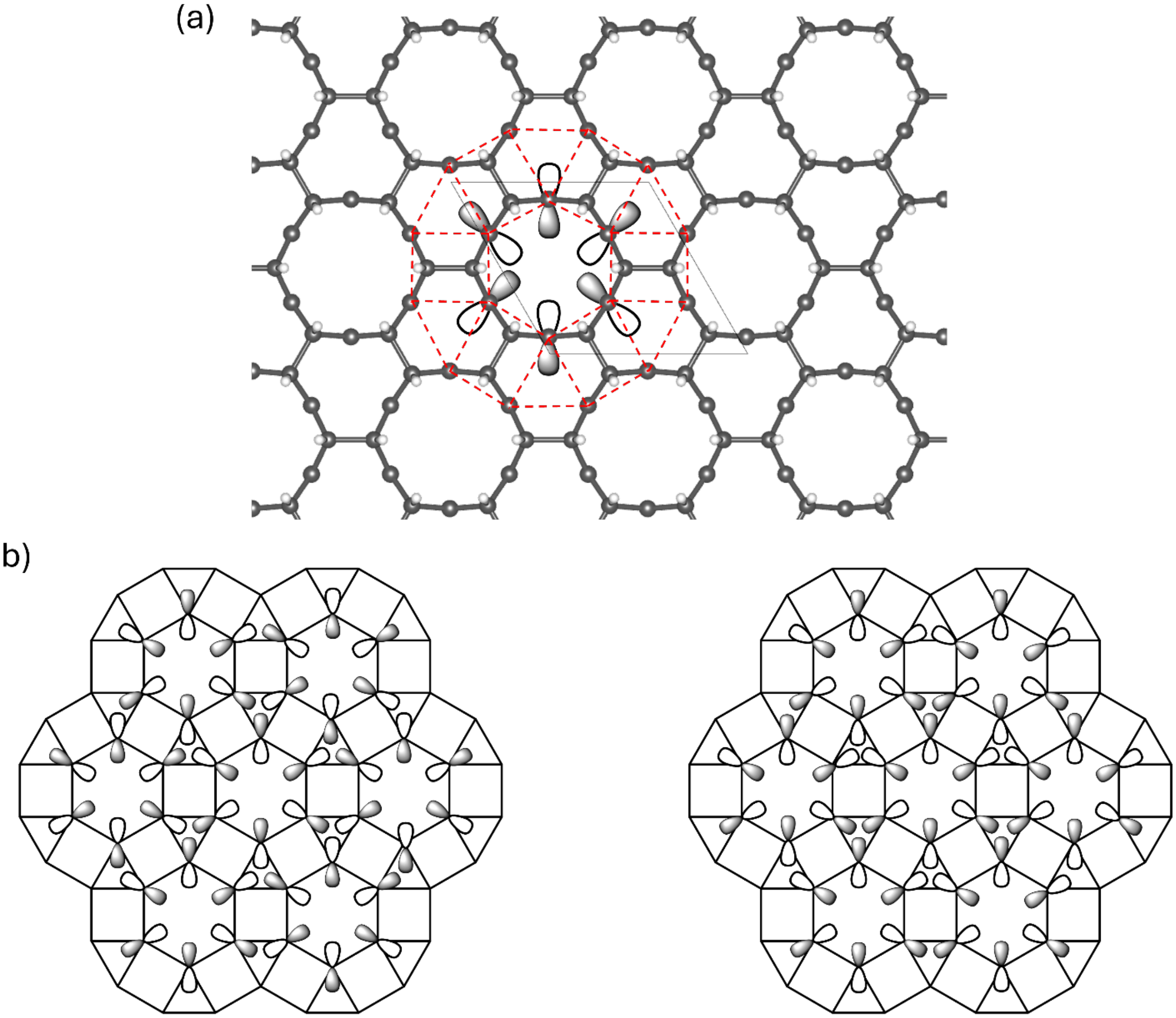

Figure S13. (a) 6-cyc-CL along with p-orbitals in the unit cell. Red dotted line shows the orbital interaction pattern in the 6-cyc-CL structures. (b) Orbital interaction resulting in an assembly of a 3.4.6.4 Archimedean tiling (equivalent to a hexagonal tungsten bronze (htb) net topology)[1] along with p orbital lobes. Grey and white ellipsoids represent negative and positive lobes of p orbitals, respectively.

Coordinates of relaxed optimized structures of n-cyc-CL (n = 6, 12, 18) taken from the output file (CONTCAR) of VASP.

1. 6-cyc-CL

```
Primitive Cell
  1.00000000000000
    7.0035313665245704    0.0000000000000000    0.0000000000000000
   -3.5017517981509498    6.0654237989728497    0.0000000000000000
   -0.0000000000000100    0.0000000000000000   30.0000000000000107
  C    H
   24    12
Direct
 0.0432437448809182  0.0995119369553308  0.5223087063844503
 0.0432275109793920  0.0995179723317463  0.4776913679455177
 0.0432663567171900  0.7002935272332849  0.4776968936848149
 0.0432857386888372  0.7002906795716513  0.5222997972436545
 0.2435107913684149  0.8839824952938713  0.5459185462869911
 0.4437462032386321  0.9005203036428782  0.5223075607144168
 0.4437310967689569  0.9005282952036850  0.4776919949009297
 0.2434663112986044  0.8839950579502514  0.4540804474019922
 0.6274223611724850  0.8839277632248752  0.5459244900530535
 0.6440042859719171  0.7002620283933751  0.5223037772753827
 0.6439787917307527  0.7002675391650186  0.4776977924035108
 0.6273763263115768  0.8839319475270599  0.4540750319260951
 0.6274988248804974  0.5000173921124258  0.5459213105831537
 0.6274495801146003  0.5000292785821614  0.4540803307071641
 0.4437301514354317  0.2997792943334474  0.4776963258090063
 0.4437540113852663  0.2997761852025818  0.5223046354350345
 0.2435075722953286  0.1161038529342164  0.5459241202812564
 0.2434645486079177  0.1161136770748925  0.4540759370435055
 0.8595429307067590  0.1160546359847814  0.5459185198572385
 0.8430135921634658  0.2997529678203392  0.5222975963245275
```

0.8429953086765494	0.2997502072785920	0.4776992988581057
0.8595104365983346	0.1160536325774544	0.4540810731424543
0.8595940545432228	0.5000230080646091	0.5459149201598592
0.8595438940427300	0.5000222858905872	0.4540818891359493
0.2435070870015110	0.8392274168097913	0.5810940711841681
0.2434408985706185	0.8392454928608615	0.4189041259931017
0.2434690981065515	0.1608504528521095	0.5810967640881088
0.5827273455748028	0.5000116946320148	0.5810862581130749
0.5826303303107920	0.8391755627999444	0.5810974600274150
0.5825653516165588	0.8391786241714314	0.4189025399725850
0.5826556937987135	0.5000326626208746	0.4189140581615334
0.2434081165689364	0.1608725657106334	0.4189027222145612
0.9043533241704083	0.5000135314904171	0.5810868787714156
0.9042950863738781	0.1608038267816525	0.5810945885233920
0.9042242171044563	0.1608099227864415	0.4189042304067456
0.9042792838591041	0.5000304384784329	0.4189118508546059

0.00000000E+00	0.00000000E+00	0.00000000E+00
0.00000000E+00	0.00000000E+00	0.00000000E+00
0.00000000E+00	0.00000000E+00	0.00000000E+00
0.00000000E+00	0.00000000E+00	0.00000000E+00
0.00000000E+00	0.00000000E+00	0.00000000E+00
0.00000000E+00	0.00000000E+00	0.00000000E+00
0.00000000E+00	0.00000000E+00	0.00000000E+00
0.00000000E+00	0.00000000E+00	0.00000000E+00
0.00000000E+00	0.00000000E+00	0.00000000E+00
0.00000000E+00	0.00000000E+00	0.00000000E+00
0.00000000E+00	0.00000000E+00	0.00000000E+00
0.00000000E+00	0.00000000E+00	0.00000000E+00
0.00000000E+00	0.00000000E+00	0.00000000E+00
0.00000000E+00	0.00000000E+00	0.00000000E+00
0.00000000E+00	0.00000000E+00	0.00000000E+00
0.00000000E+00	0.00000000E+00	0.00000000E+00
0.00000000E+00	0.00000000E+00	0.00000000E+00
0.00000000E+00	0.00000000E+00	0.00000000E+00

0.00000000E+00 0.00000000E+00 0.00000000E+00
0.00000000E+00 0.00000000E+00 0.00000000E+00
0.00000000E+00 0.00000000E+00 0.00000000E+00
0.00000000E+00 0.00000000E+00 0.00000000E+00
0.00000000E+00 0.00000000E+00 0.00000000E+00
0.00000000E+00 0.00000000E+00 0.00000000E+00
0.00000000E+00 0.00000000E+00 0.00000000E+00
0.00000000E+00 0.00000000E+00 0.00000000E+00
0.00000000E+00 0.00000000E+00 0.00000000E+00
0.00000000E+00 0.00000000E+00 0.00000000E+00
0.00000000E+00 0.00000000E+00 0.00000000E+00
0.00000000E+00 0.00000000E+00 0.00000000E+00
0.00000000E+00 0.00000000E+00 0.00000000E+00
0.00000000E+00 0.00000000E+00 0.00000000E+00
0.00000000E+00 0.00000000E+00 0.00000000E+00
0.00000000E+00 0.00000000E+00 0.00000000E+00
0.00000000E+00 0.00000000E+00 0.00000000E+00
0.00000000E+00 0.00000000E+00 0.00000000E+00

2. 12-cyc-CL

Primitive Cell
1.00000000000000
11.8664980944054843 0.0000170233597039 0.0169497434898908
5.9303255530786405 10.2775964810420284 0.1185029721932685
0.0000000000000000 0.0000000000000000 30.0000000000000000
C H
48 24
Direct
0.4205671592466729 0.6308869921235143 0.5473123885133973
0.3181511183695065 0.7350243045152780 0.5238293720621598
0.2100204942957546 0.8521286525850087 0.5472192783330385
0.3172815726036049 0.7383087725312620 0.4760565444792206
0.2078016604551820 0.8581484525857235 0.4527433126162563
0.1987548241187227 0.9751293117678941 0.4761292856736929
0.2002066437805752 0.9719175541309468 0.5239034254416950

0.5230422800944865	0.5307375669413119	0.5238474542111859
0.4191803292369798	0.6376805517613917	0.4525605918313502
0.1974927082117048	0.0792311250041209	0.4526454286766821
0.2009068918078185	0.0726997680013355	0.5473963439362990
0.5221586006581518	0.5341675382749145	0.4760732749987113
0.2006833204223781	0.1762714252061244	0.5239051748129242
0.1990362195780975	0.1796229279698096	0.4761254529628971
0.9637476170579333	0.4153768533241973	0.4760878952264420
0.9653588608970622	0.4116283224862372	0.5238621248533732
0.8605885909130251	0.4175153395517555	0.4525884193323861
0.8639186292770731	0.4094739652427251	0.5473296245834476
0.7603420916003643	0.4122255230193090	0.5238573209360595
0.7589004983137784	0.4160078594197998	0.4760929677980528
0.6400317433322158	0.4269846801563659	0.4527270574449318
0.6422441414459854	0.4205111322865847	0.5471838645168532
0.6423520419617219	0.2842729543886335	0.5473055056392653
0.7601328636219478	0.1770051867459514	0.5239274604838613
0.8628024585292877	0.0731682940475551	0.5474183412314986
0.7592333199604937	0.1804066357669072	0.4761563915300329
0.8613749077189397	0.0799346265155023	0.4526866839558465
0.9635460423818643	0.9754987492970741	0.4761620766154167
0.9644161686759318	0.9722447711731022	0.5239244984132796
0.5235418776444618	0.2950312683491703	0.5239522021775755
0.6400894161601869	0.2907513510804947	0.4528365345267673
0.5220917939224634	0.2988151033720996	0.4761760068653729
0.2082286669128308	0.2899711747958946	0.4528209434099892
0.2110307180899014	0.2835032902328152	0.5472721343278977
0.3192885077530505	0.2944815543115240	0.5239288794347630
0.3176603551756614	0.2982904862525757	0.4761640183630078
0.4187970164376438	0.3010781553335278	0.4526993819500404
0.4221596917728476	0.2929211025953151	0.5474442287298942
0.0829960385487638	0.7352731680400595	0.5238858253464969
0.0815620429920827	0.7384566418486358	0.4761233171687707
0.0847858231676994	0.6308878386326526	0.5473636056053195
0.0839151015800397	0.5301710379229263	0.5238833939942253

0.0749368514163109	0.4197090192822657	0.5472010548769077
0.0822724121809912	0.5335145085836928	0.4761127515627237
0.0721225128522391	0.4261855548727382	0.4527361249155604
0.0814256131547211	0.6373928007270635	0.4526314141427648
0.0715518628525658	0.8582953616897484	0.4527939237434566
0.0737329302733087	0.8523571169582951	0.5472472383148954
0.2329174434372163	0.8602606011830858	0.4168484555209488
0.1983347448791548	0.0804592274277738	0.4154666631123121
0.2049432076959619	0.0689921125079849	0.5845720746681877
0.4218792807503959	0.6293987057632222	0.5844939643107807
0.2368703521863722	0.8499392221977118	0.5830992427832484
0.4201231319211089	0.6414659720898186	0.4153867600995582
0.8640883455184394	0.4087061682017037	0.5845054789042194
0.8577743854356967	0.4236013321796861	0.4154165392652303
0.6392742093663770	0.4550292092919079	0.4168165931908732
0.6429910152798399	0.4444666786972178	0.5830451872530347
0.8599514864928111	0.0814206764137779	0.4155047301789025
0.6431870248079932	0.2561747058940824	0.5832136561140828
0.8618881037877770	0.0693922903368218	0.5845953691419226
0.6392793939738866	0.2668467978788698	0.4169738596726020
0.2378610525915121	0.2553639002945260	0.5831717794378122
0.2333681642240251	0.2660951879207545	0.4169505764787758
0.4185918453366142	0.3020162968771274	0.4155213065764798
0.4249107377957557	0.2868808700938246	0.5846128830066561
0.0838044353366953	0.6297137456822952	0.5845447949524711
0.0774110757541067	0.6410698216415085	0.4154547220691640
0.0453283195359830	0.4543031204103514	0.4168328667232046
0.0498384653425674	0.4435415506948246	0.5830762784892940
0.0446531067580409	0.8604262924064230	0.4169213638222971
0.0484969493365526	0.8503174312128081	0.5831360736392881

0.00000000E+00	0.00000000E+00	0.00000000E+00
0.00000000E+00	0.00000000E+00	0.00000000E+00
0.00000000E+00	0.00000000E+00	0.00000000E+00
0.00000000E+00	0.00000000E+00	0.00000000E+00

0.00000000E+00	0.00000000E+00	0.00000000E+00
0.00000000E+00	0.00000000E+00	0.00000000E+00
0.00000000E+00	0.00000000E+00	0.00000000E+00
0.00000000E+00	0.00000000E+00	0.00000000E+00
0.00000000E+00	0.00000000E+00	0.00000000E+00
0.00000000E+00	0.00000000E+00	0.00000000E+00
0.00000000E+00	0.00000000E+00	0.00000000E+00
0.00000000E+00	0.00000000E+00	0.00000000E+00
0.00000000E+00	0.00000000E+00	0.00000000E+00
0.00000000E+00	0.00000000E+00	0.00000000E+00
0.00000000E+00	0.00000000E+00	0.00000000E+00
0.00000000E+00	0.00000000E+00	0.00000000E+00
0.00000000E+00	0.00000000E+00	0.00000000E+00
0.00000000E+00	0.00000000E+00	0.00000000E+00
0.00000000E+00	0.00000000E+00	0.00000000E+00
0.00000000E+00	0.00000000E+00	0.00000000E+00
0.00000000E+00	0.00000000E+00	0.00000000E+00
0.00000000E+00	0.00000000E+00	0.00000000E+00
0.00000000E+00	0.00000000E+00	0.00000000E+00
0.00000000E+00	0.00000000E+00	0.00000000E+00
0.00000000E+00	0.00000000E+00	0.00000000E+00
0.00000000E+00	0.00000000E+00	0.00000000E+00
0.00000000E+00	0.00000000E+00	0.00000000E+00
0.00000000E+00	0.00000000E+00	0.00000000E+00
0.00000000E+00	0.00000000E+00	0.00000000E+00
0.00000000E+00	0.00000000E+00	0.00000000E+00
0.00000000E+00	0.00000000E+00	0.00000000E+00
0.00000000E+00	0.00000000E+00	0.00000000E+00
0.00000000E+00	0.00000000E+00	0.00000000E+00
0.00000000E+00	0.00000000E+00	0.00000000E+00
0.00000000E+00	0.00000000E+00	0.00000000E+00
0.00000000E+00	0.00000000E+00	0.00000000E+00
0.00000000E+00	0.00000000E+00	0.00000000E+00
0.00000000E+00	0.00000000E+00	0.00000000E+00
0.00000000E+00	0.00000000E+00	0.00000000E+00

0.00000000E+00	0.00000000E+00	0.00000000E+00
0.00000000E+00	0.00000000E+00	0.00000000E+00
0.00000000E+00	0.00000000E+00	0.00000000E+00
0.00000000E+00	0.00000000E+00	0.00000000E+00
0.00000000E+00	0.00000000E+00	0.00000000E+00
0.00000000E+00	0.00000000E+00	0.00000000E+00
0.00000000E+00	0.00000000E+00	0.00000000E+00
0.00000000E+00	0.00000000E+00	0.00000000E+00
0.00000000E+00	0.00000000E+00	0.00000000E+00
0.00000000E+00	0.00000000E+00	0.00000000E+00
0.00000000E+00	0.00000000E+00	0.00000000E+00
0.00000000E+00	0.00000000E+00	0.00000000E+00
0.00000000E+00	0.00000000E+00	0.00000000E+00
0.00000000E+00	0.00000000E+00	0.00000000E+00
0.00000000E+00	0.00000000E+00	0.00000000E+00
0.00000000E+00	0.00000000E+00	0.00000000E+00
0.00000000E+00	0.00000000E+00	0.00000000E+00
0.00000000E+00	0.00000000E+00	0.00000000E+00
0.00000000E+00	0.00000000E+00	0.00000000E+00
0.00000000E+00	0.00000000E+00	0.00000000E+00
0.00000000E+00	0.00000000E+00	0.00000000E+00
0.00000000E+00	0.00000000E+00	0.00000000E+00
0.00000000E+00	0.00000000E+00	0.00000000E+00
0.00000000E+00	0.00000000E+00	0.00000000E+00
0.00000000E+00	0.00000000E+00	0.00000000E+00
0.00000000E+00	0.00000000E+00	0.00000000E+00
0.00000000E+00	0.00000000E+00	0.00000000E+00
0.00000000E+00	0.00000000E+00	0.00000000E+00
0.00000000E+00	0.00000000E+00	0.00000000E+00
0.00000000E+00	0.00000000E+00	0.00000000E+00
0.00000000E+00	0.00000000E+00	0.00000000E+00
0.00000000E+00	0.00000000E+00	0.00000000E+00
0.00000000E+00	0.00000000E+00	0.00000000E+00

3. 18-cyc-CL

Primitive Cell

1.00000000000000

16.7716109928686983 -0.0006411577037985 -0.0004697823061846

-8.3848328302309625 14.5273276479577298 -0.0005518907992345

-0.0008403257148333 -0.0016248256040499 29.9902815543379475

C H

72 36

Direct

0.1139244949733680 0.2904229922066150 0.5464855661860213

0.1843651060381077 0.2887870372431820 0.5235999745003994

0.2614333523133610 0.2821546688246315 0.5463885284223622

0.1843083359252233 0.2885939676003915 0.4760156398246593

0.2616213396868829 0.2819994948508722 0.4534874209969431

0.2681467799630397 0.2048271234562904 0.4762558126317771

0.2682836892705112 0.2049696527181482 0.5238414235981423

0.0404234377236463 0.2916952458054070 0.5236841756345996

0.1137490585747400 0.2899719758614978 0.4530525784474833

0.2693224862554757 0.1340590383987958 0.4534302638074763

0.2698880837838544 0.1345524776723508 0.5468574385555343

0.0402856454924247 0.2914595637701893 0.4758407761885906

0.2710821208388623 0.0608856268007969 0.5241384774912206

0.2706789542672325 0.0606494181000130 0.4762934645289505

0.5054430051723884 0.9860343029219933 0.5468857069364645

0.5789181542102497 0.0609741108375701 0.5241335668713205

0.6537965117960596 0.1345306579393437 0.5469100488122578

0.5789287910533716 0.0609686243198411 0.4762903723447636

0.6536908013352374 0.1344227304669019 0.4534903928928458

0.7258029786712044 0.2048575230307534 0.4763714433967792

0.7258960162642012 0.2049405792985723 0.5239587254857554

0.5055377800530536 0.9861464875125847 0.4534529625925501

0.8094695143326192 0.2819320296176215 0.4535739311787736

0.8098382625457887 0.2822189621113154 0.5464958770040553

0.8935825756977920 0.2888301667128275 0.5237021936748008

0.8934249137910371	0.2886546754052048	0.4761298195798460
0.9653327227225148	0.2900707696289544	0.4531186330283745
0.9656824056746274	0.2903785925626394	0.5465450515534300
0.6537674713781030	0.3699980511101515	0.5468209018370871
0.5788606770184188	0.3686327841264330	0.5240675596842834
0.5053514598527187	0.3700858059660490	0.5468272650080905
0.5788194874939521	0.3685483808600551	0.4762301669209208
0.5054032585406389	0.3700167301674782	0.4534094535645465
0.4349730968420786	0.3717554023230605	0.4762701181942575
0.4349609374203496	0.3717895149549406	0.5238629887693804
0.7258309616570884	0.3716490628915849	0.5238550820380610
0.6536065086002409	0.3698516308908140	0.4534103836469470
0.7257686426791565	0.3715566090164444	0.4762787623489118
0.2698569258883268	0.9860539952585441	0.5469345817506337
0.1136934772108447	0.6744311751745542	0.5466068514614264
0.1140313796565664	0.6743630323995689	0.4531866263580469
0.0404531449251877	0.5994424426671898	0.4759222782167285
0.0402629718653955	0.5996111492251828	0.5237615376944049
0.2691943965793939	0.9857520464695781	0.4535038302828340
0.9656791030117446	0.5258051759313318	0.4531427117423661
0.9654266872087405	0.5261928384993411	0.5465541275543728
0.8934347752829126	0.4556134936088583	0.5236749433479915
0.8935017530302645	0.4554668706074736	0.4760966274804730
0.8095385788337879	0.3782246422786812	0.4535202955498931
0.8097788245652211	0.3784589742254099	0.5464495045861213
0.4349830181906071	0.9141631231620124	0.4763204696916645
0.4349873363387644	0.9140935358456304	0.5239021145828460
0.1844551068640214	0.7463267654888384	0.4761785627459716
0.2615383515058696	0.8302781344626311	0.4536177435190965
0.2680556404410623	0.9139301655535021	0.4763978164270455
0.2682158227300988	0.9139714172814877	0.5239929836106256
0.2615735941872472	0.8300581184988971	0.5465352405613046
0.1843928451681194	0.7463439862639873	0.5237604631928505
0.3578283976624306	0.3783353154619959	0.4535251223016914
0.3577046754111493	0.3784422299445112	0.5464549790027462

0.3579343904089640	0.8304219528163159	0.4535638565993594
0.3579190943128125	0.8301671194288929	0.5464779969084148
0.3510135001897225	0.4554552053945002	0.4761195679696542
0.3494675104744758	0.5258991892204250	0.4531388862095369
0.3497553096347730	0.5261713870449043	0.5465710003748185
0.3511166969589681	0.4555626877985688	0.5237173678205025
0.3483769458211725	0.5996180314248463	0.4758929102294189
0.3498789861610518	0.6745840291886935	0.4531292419511388
0.3513699706073510	0.7465570795928329	0.4761132725249908
0.3514160572677696	0.7465081996246568	0.5236980062929462
0.3500564107456938	0.6745346182450618	0.5465606982458411
0.3485531662647645	0.5996896164241008	0.5237277264224218
0.2436237605434002	0.2637712698604133	0.4182765476191861
0.2679347944116870	0.1331402435825098	0.4169870849893442
0.2690306010130357	0.1341938962169920	0.5833242244012524
0.1133108764543280	0.2895708853998258	0.5829288343164549
0.2430739893953131	0.2639377417571822	0.5815627833304191
0.1130118563338556	0.2886868596750531	0.4165916036970998
0.5060415374737772	0.9854084478829037	0.5833424680053696
0.5063631332637755	0.9856029172894125	0.4170109271726190
0.6539729315926337	0.1337818850531818	0.4170384911191434
0.6541352800278162	0.1339500996500433	0.5833718266834467
0.8101318658884793	0.2642283725664853	0.5816986956066117
0.8093145742591901	0.2635376748292018	0.4184049421040754
0.9645858132404044	0.2887698349997123	0.4166543814551815
0.9653503620230950	0.2892467697763266	0.5829954125618286
0.5060088689348987	0.3708986891579187	0.4169618915238544
0.6543133981843638	0.3710833560203994	0.5832784646555709
0.5058558918777365	0.3710278387101127	0.5832904278524538
0.6539295859007481	0.3707559392688253	0.4169478726808507
0.2690265077227849	0.9857442282312974	0.5834028009541612
0.2677487124983386	0.9851603173633663	0.4170673811375565
0.1135509698761368	0.6749952236471088	0.4167312084153139
0.1127981975894556	0.6749394926739924	0.5830557269033108
0.9650115989376928	0.5269863401916268	0.5830034461215234

0.9655021626790230	0.5262046226161914	0.4166738903215474
0.8093276127216669	0.3961680936012399	0.4183094459874042
0.8098873768653405	0.3967171888554830	0.5816288641942364
0.2433967903637182	0.8303890078342491	0.4184239530654210
0.2433652305424445	0.8297458627919525	0.5817530603860135
0.3760581060410573	0.3965626646645575	0.4183401906138968
0.3756592779956236	0.3965520009542729	0.5816647624073639
0.3761171619436467	0.8306154807258181	0.4183494144220106
0.3503400208216760	0.5263599359636046	0.4166702137335392
0.3508431461333856	0.5269374216509704	0.5830187469676318
0.3510183224558148	0.6751461173123943	0.4166650079327283
0.3513005083524092	0.6749488229480676	0.5830033932658623
0.3761364390734343	0.8300199360352494	0.5816556896402219

0.00000000E+00	0.00000000E+00	0.00000000E+00
0.00000000E+00	0.00000000E+00	0.00000000E+00
0.00000000E+00	0.00000000E+00	0.00000000E+00
0.00000000E+00	0.00000000E+00	0.00000000E+00
0.00000000E+00	0.00000000E+00	0.00000000E+00
0.00000000E+00	0.00000000E+00	0.00000000E+00
0.00000000E+00	0.00000000E+00	0.00000000E+00
0.00000000E+00	0.00000000E+00	0.00000000E+00
0.00000000E+00	0.00000000E+00	0.00000000E+00
0.00000000E+00	0.00000000E+00	0.00000000E+00
0.00000000E+00	0.00000000E+00	0.00000000E+00
0.00000000E+00	0.00000000E+00	0.00000000E+00
0.00000000E+00	0.00000000E+00	0.00000000E+00
0.00000000E+00	0.00000000E+00	0.00000000E+00
0.00000000E+00	0.00000000E+00	0.00000000E+00
0.00000000E+00	0.00000000E+00	0.00000000E+00
0.00000000E+00	0.00000000E+00	0.00000000E+00
0.00000000E+00	0.00000000E+00	0.00000000E+00
0.00000000E+00	0.00000000E+00	0.00000000E+00
0.00000000E+00	0.00000000E+00	0.00000000E+00
0.00000000E+00	0.00000000E+00	0.00000000E+00

0.00000000E+00	0.00000000E+00	0.00000000E+00
0.00000000E+00	0.00000000E+00	0.00000000E+00
0.00000000E+00	0.00000000E+00	0.00000000E+00
0.00000000E+00	0.00000000E+00	0.00000000E+00
0.00000000E+00	0.00000000E+00	0.00000000E+00
0.00000000E+00	0.00000000E+00	0.00000000E+00
0.00000000E+00	0.00000000E+00	0.00000000E+00
0.00000000E+00	0.00000000E+00	0.00000000E+00
0.00000000E+00	0.00000000E+00	0.00000000E+00
0.00000000E+00	0.00000000E+00	0.00000000E+00
0.00000000E+00	0.00000000E+00	0.00000000E+00
0.00000000E+00	0.00000000E+00	0.00000000E+00
0.00000000E+00	0.00000000E+00	0.00000000E+00
0.00000000E+00	0.00000000E+00	0.00000000E+00
0.00000000E+00	0.00000000E+00	0.00000000E+00
0.00000000E+00	0.00000000E+00	0.00000000E+00
0.00000000E+00	0.00000000E+00	0.00000000E+00
0.00000000E+00	0.00000000E+00	0.00000000E+00
0.00000000E+00	0.00000000E+00	0.00000000E+00
0.00000000E+00	0.00000000E+00	0.00000000E+00
0.00000000E+00	0.00000000E+00	0.00000000E+00
0.00000000E+00	0.00000000E+00	0.00000000E+00
0.00000000E+00	0.00000000E+00	0.00000000E+00
0.00000000E+00	0.00000000E+00	0.00000000E+00
0.00000000E+00	0.00000000E+00	0.00000000E+00
0.00000000E+00	0.00000000E+00	0.00000000E+00
0.00000000E+00	0.00000000E+00	0.00000000E+00
0.00000000E+00	0.00000000E+00	0.00000000E+00
0.00000000E+00	0.00000000E+00	0.00000000E+00
0.00000000E+00	0.00000000E+00	0.00000000E+00
0.00000000E+00	0.00000000E+00	0.00000000E+00
0.00000000E+00	0.00000000E+00	0.00000000E+00
0.00000000E+00	0.00000000E+00	0.00000000E+00
0.00000000E+00	0.00000000E+00	0.00000000E+00
0.00000000E+00	0.00000000E+00	0.00000000E+00

0.00000000E+00	0.00000000E+00	0.00000000E+00
0.00000000E+00	0.00000000E+00	0.00000000E+00
0.00000000E+00	0.00000000E+00	0.00000000E+00
0.00000000E+00	0.00000000E+00	0.00000000E+00
0.00000000E+00	0.00000000E+00	0.00000000E+00
0.00000000E+00	0.00000000E+00	0.00000000E+00
0.00000000E+00	0.00000000E+00	0.00000000E+00
0.00000000E+00	0.00000000E+00	0.00000000E+00
0.00000000E+00	0.00000000E+00	0.00000000E+00
0.00000000E+00	0.00000000E+00	0.00000000E+00
0.00000000E+00	0.00000000E+00	0.00000000E+00
0.00000000E+00	0.00000000E+00	0.00000000E+00
0.00000000E+00	0.00000000E+00	0.00000000E+00
0.00000000E+00	0.00000000E+00	0.00000000E+00
0.00000000E+00	0.00000000E+00	0.00000000E+00
0.00000000E+00	0.00000000E+00	0.00000000E+00
0.00000000E+00	0.00000000E+00	0.00000000E+00
0.00000000E+00	0.00000000E+00	0.00000000E+00
0.00000000E+00	0.00000000E+00	0.00000000E+00
0.00000000E+00	0.00000000E+00	0.00000000E+00
0.00000000E+00	0.00000000E+00	0.00000000E+00
0.00000000E+00	0.00000000E+00	0.00000000E+00
0.00000000E+00	0.00000000E+00	0.00000000E+00
0.00000000E+00	0.00000000E+00	0.00000000E+00
0.00000000E+00	0.00000000E+00	0.00000000E+00
0.00000000E+00	0.00000000E+00	0.00000000E+00
0.00000000E+00	0.00000000E+00	0.00000000E+00
0.00000000E+00	0.00000000E+00	0.00000000E+00
0.00000000E+00	0.00000000E+00	0.00000000E+00
0.00000000E+00	0.00000000E+00	0.00000000E+00
0.00000000E+00	0.00000000E+00	0.00000000E+00
0.00000000E+00	0.00000000E+00	0.00000000E+00
0.00000000E+00	0.00000000E+00	0.00000000E+00
0.00000000E+00	0.00000000E+00	0.00000000E+00
0.00000000E+00	0.00000000E+00	0.00000000E+00

0.00000000E+00	0.00000000E+00	0.00000000E+00
0.00000000E+00	0.00000000E+00	0.00000000E+00
0.00000000E+00	0.00000000E+00	0.00000000E+00
0.00000000E+00	0.00000000E+00	0.00000000E+00
0.00000000E+00	0.00000000E+00	0.00000000E+00
0.00000000E+00	0.00000000E+00	0.00000000E+00
0.00000000E+00	0.00000000E+00	0.00000000E+00
0.00000000E+00	0.00000000E+00	0.00000000E+00
0.00000000E+00	0.00000000E+00	0.00000000E+00
0.00000000E+00	0.00000000E+00	0.00000000E+00
0.00000000E+00	0.00000000E+00	0.00000000E+00
0.00000000E+00	0.00000000E+00	0.00000000E+00
0.00000000E+00	0.00000000E+00	0.00000000E+00
0.00000000E+00	0.00000000E+00	0.00000000E+00
0.00000000E+00	0.00000000E+00	0.00000000E+00
0.00000000E+00	0.00000000E+00	0.00000000E+00
0.00000000E+00	0.00000000E+00	0.00000000E+00

Reference

1. S.-L. Cai, Z.-H. He, X.-L. Li, K. Zhang, S.-R. Zheng, J. Fan, Y. Liu and W.-G. Zhang, *Chemical Communications*, 2019, **55**, 13454-13457.
